# Artificial intelligence support improves diagnosis accuracy in anterior segment eye diseases

**DOI:** 10.1038/s41598-025-89768-6

**Published:** 2025-02-11

**Authors:** Hiroki Maehara, Yuta Ueno, Takefumi Yamaguchi, Yoshiyuki Kitaguchi, Dai Miyazaki, Ryohei Nejima, Takenori Inomata, Naoko Kato, Tai-ichiro Chikama, Jun Ominato, Tatsuya Yunoki, Kinya Tsubota, Masahiro Oda, Manabu Suzutani, Tetsuju Sekiryu, Tetsuro Oshika

**Affiliations:** 1 Department of Ophthalmology, Fukushima Medical University School of Medicine, Fukushima, Japan; 2 Departement of Ophthalmology, Faculty of Medicine, University of Tsukuba, 1-1-1 Tennoudai, Tsukuba, Ibaraki 305-8576 Japan; 3 Department of Ophthalmology, Tokyo Dental College Ichikawa General Hospital, Chiba, Japan; 4 Department of Ophthalmology, Osaka University Graduate School of Medicine, Osaka, Japan; 5 Division of Ophthalmology and Visual Science, Faculty of Medicine, Tottori University, Tottori, Japan; 6 Department of Ophthalmology, Miyata Eye Hospital, Miyazaki, Japan; 7 Department of Ophthalmology, Juntendo University Graduate School of Medicine, Tokyo, Japan; 8 Department of Ophthalmology, Tsukazaki Hospital, Hyogo, Japan; 9 Division of Ophthalmology and Visual Science, Graduate School of Biomedical and Health Sciences, Hiroshima University, Hiroshima, Japan; 10 Division of Ophthalmology and Visual Science, Graduate School of Medical and Dental Sciences, Niigata University, Niigata, Japan; 11 Department of Ophthalmology, University of Toyama, Toyama, Japan; 12 Department of Ophthalmology, Tokyo Medical University, Tokyo, Japan; 13 Graduate School of Informatics, Nagoya University, Nagoya, Japan; 14 Japan Anterior Segment Artificial Intelligence Research Group, Tsukuba, Japan

**Keywords:** Artificial intelligence, Ocular surface, AI support, Smartphone image, Slit-lamp image, Corneal diseases, Diagnosis

## Abstract

CorneAI, a deep learning model designed for diagnosing cataracts and corneal diseases, was assessed for its impact on ophthalmologists’ diagnostic accuracy. In the study, 40 ophthalmologists (20 specialists and 20 residents) classified 100 images, including iPhone 13 Pro photos (50 images) and diffuser slit-lamp photos (50 images), into nine categories (normal condition, infectious keratitis, immunological keratitis, corneal scar, corneal deposit, bullous keratopathy, ocular surface tumor, cataract/intraocular lens opacity, and primary angle-closure glaucoma). The iPhone and slit-lamp images represented the same cases. After initially answering without CorneAI, the same ophthalmologists responded to the same cases with CorneAI 2–4 weeks later. With CorneAI’s support, the overall accuracy of ophthalmologists increased significantly from 79.2 to 88.8% (*P* < 0.001). Specialists’ accuracy rose from 82.8 to 90.0%, and residents’ from 75.6 to 86.2% (*P* < 0.001). Smartphone image accuracy improved from 78.7 to 85.5% and slit-lamp image accuracy from 81.2 to 90.6% (both, *P* < 0.001). In this study, CorneAI’s own accuracy was 86%, but its support enhanced ophthalmologists’ accuracy beyond the CorneAI’s baseline. This study demonstrated that CorneAI, despite being trained on diffuser slit-lamp images, effectively improved diagnostic accuracy, even with smartphone images.

## Introduction

Telemedicine can be particularly useful in the field of ophthalmology, where some conditions can be diagnosed and managed remotely^1^. In recent years, artificial intelligence (AI) has been used to assist in the diagnosis and management of various medical conditions, including ophthalmic diseases such as infectious keratitis^2,3^. AI algorithms have been developed to analyze fundus images and provide potential diagnoses or recommend patients for further evaluation by ophthalmologists. Ocular imaging plays a crucial role in diagnosing and managing various anterior segment disorders. We developed an AI-based classification tool, CorneAI, using a dataset of anterior segment color images taken by slit-lamp microscopy using diffuser light^4^. It classifies various corneal conditions into 9 categories (infectious keratitis, immunological keratitis, scarring, corneal deposit dystrophy, bullous keratopathy, ocular surface tumor, cataract/IOL (intra ocular lens) opacity, primary angle-closure glaucoma, and normal condition) with sufficient performance exceeding that of board-certified specialists on the basis of images taken by both slit-lamp microscope and smartphone cameras (Table S1)^4^. CorneAI was developed using 5270 slit-lamp microscopy images collected from 18 institutions affiliated with the Japan Cornea Society. All images were meticulously verified by four corneal specialists, who validated diagnoses made by tertiary centers. They classified corneal diseases and cataracts into nine categories that encompass the major anterior segment diseases of the eye. We employed You Only Look Once (YOLO) Version 5 (YOLO V.5) as the AI algorithm to perform the nine-category classification. The model parameters in YOLO V.5 were pretrained using the Common Objects in Context dataset, followed by fine-tuning with the training dataset. YOLO V.5 was trained for 200 epochs with a mini-batch size of 16. The YOLO V.5 model achieved an area under the curve (AUC) ranging from 0.931 to 0.998, a sensitivity of 0.628–0.958, and a specificity of 0.969–0.998^4^. In light of the rapidly evolving technology, it is essential to demonstrate to ophthalmologists the potential for utilizing simple and effective smartphone imaging techniques. Smartphone-based imaging has already been introduced for screening purposes for retinopathy of prematurity^5^, and anterior segment diseases imaging attached to a slit-lamp microscope^6,7^ or using special attachments^8–10^. In the future, combination of smartphone images and AI technologies can significantly streamline our practices by teleconsultations, providing diagnoses and advice from senior medical experts to local clinics or patients.

We expect that CorneAI will improve diagnostic accuracy by ophthalmologists in clinics, facilitate patients to visit hospitals at the early stages of the diseases in local communities, by presenting potential diagnoses. However, the impact of AI-assisted diagnosis in corneal diseases is not yet fully understood. In addition, if a patient is seen in an emergency at a medical facility that does not have an ophthalmologist, CorneAI may be able to provide an initial response. In this study, we hypothesized that CorneAI assistance can improve efficiency and accuracy of diagnosing anterior segment eye diseases by ophthalmologists. We aimed to evaluate the diagnostic performance of anterior segment diseases based on smartphone and slit-lamp images with or without CorneAI assistance.

## Materials and methods

### Subject

The research was conducted in accordance with the tenets of the Declaration of Helsinki, and all patients provided written informed consent after they received a detailed explanation of the study protocols and possible consequences associated with participation. The Institutional Review Board of University of Tsukuba, Ibaraki, Japan, approved this prospective study protocol (ID: R3-108). This study is multicenter collaborative prospective research. Smartphone and slit-lamp images were collected from 13 collaborating facilities.

### Image acquisition

**Fig. 1:**
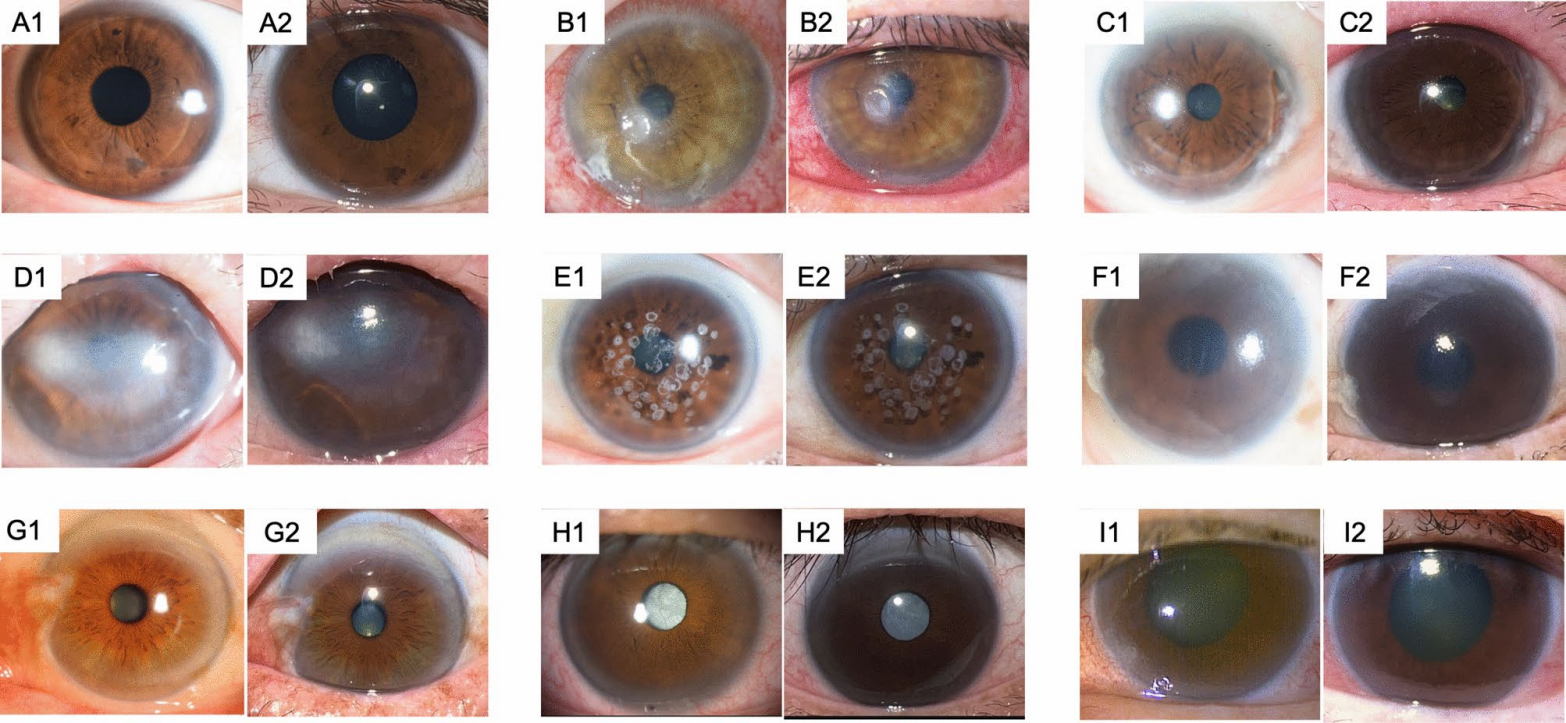
Cases of the 9 classified conditions, which include slit-lamp images and smartphone images. “1” represents the slit lamp microscope image, and “2” represents the smartphone image. (**A**) Normal condition, (**B**) Infectious keratitis, (**C**) immunological keratitis, (**D**) Corneal scarring, (**E**) Corneal deposit, (**F**) Bullous keratopathy, (**G**) Ocular surface tumor, (**H**) Cataract/intra ocular lens opacity, (**I**) Primary angle-closure glaucoma.

**Fig. 2:**
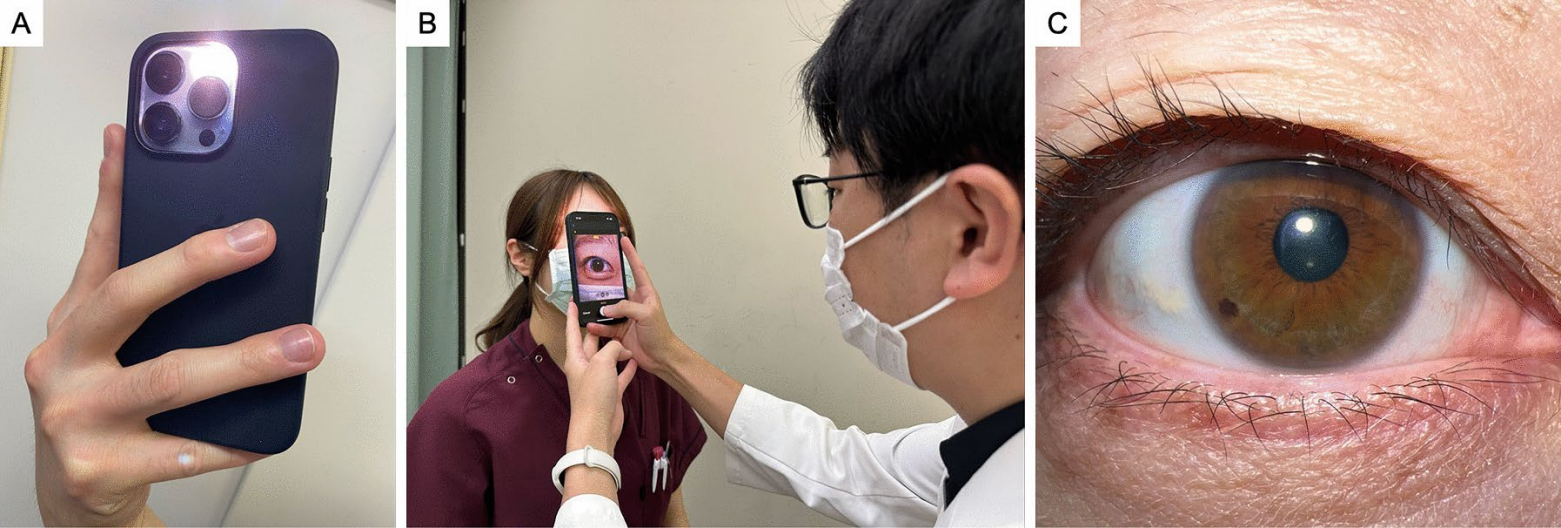
Smartphone, the capturing scene, and the captured images. (**A**) The smartphone used was an iPhone 13 Pro, and the images were taken without any attachments using flash. (**B**) Actual capturing scene. (**C**) Image of a normal condition taken with a smartphone.

We collected anterior segment images of the 9 classified conditions (Fig. 1) using slit-lamp and smartphone cameras taken on the same day. Smartphone images were captured using iPhone 13 Pro (Apple Inc., Cupertino, CA, USA) in all cases. We utilized the Ultra-Wide mode of the iPhone 13 Pro using custom-built software to display a circular guide for image capture without any additional equipment (Fig. 2). The distance from the patient’s eye was approximately 5 cm. Slit-lamp images with diffuser light were taken at × 10 or × 16 magnifications in the darkroom, ensuring that at least the entire cornea was captured in a single image.

The study population comprised 807 cases collected from 13 facilities between January and December 2022. One smartphone image and one slit-lamp image were collected for each case, resulting in a total of 1614 images (807 cases) were diagnosed by corneal specialists at each institution based on the patients’ medical histories and clinical examination data included in this cohort. Three corneal specialists (H.M, Y.U, and T.Y) examined 807 cases. Among the cases with unanimous agreement in the diagnoses among three experts, 50 cases were randomly selected (6 infectious keratitis, 5 immunological keratitis, 4 corneal scar, 7 corneal deposit dystrophy, 5 bullous keratopathy, 6 ocular surface tumor, 6 cataract/IOL opacity, and 4 primary glaucoma attacks, and 7 normal condition cases) as reported previously^4^.

### Ophthalmologist’s means of answering

**Fig. 3:**
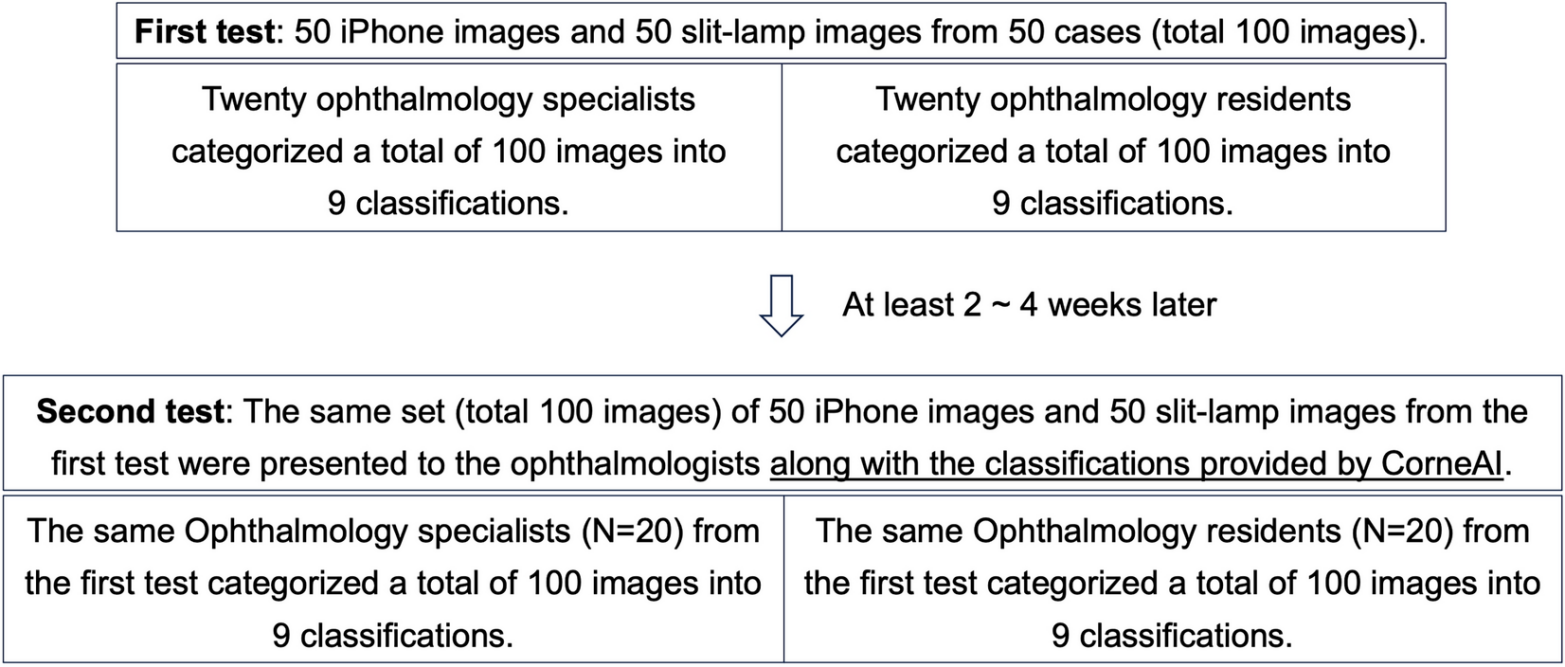
The scheme of this study. This is the scheme for the two tests conducted in this study. Both the first and second tests were administered to the same ophthalmology specialists and the same residents, with each test consisting of 100 images. In the first test, only the images were presented to the ophthalmologists. At least 2–4 weeks later, the second test was administered, this time with the diagnoses provided by CorneAI included.

**Fig. 4:**
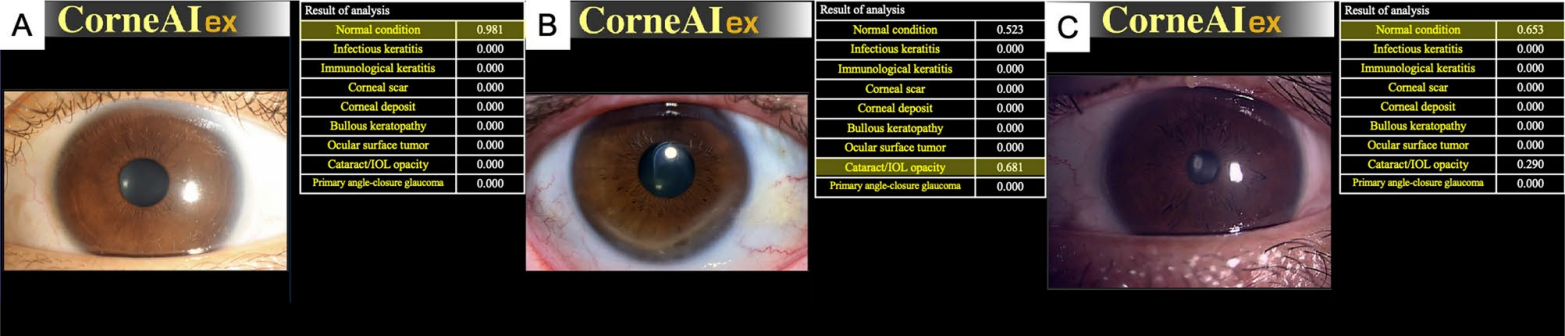
Analysis results of anterior segment diseases with CorneAI. When anterior segment images are input into CorneAI, they can be classified into nine categories (infectious keratitis, immunological keratitis, scarring, corneal deposit, bullous keratopathy, ocular surface tumor, cataract/IOL (intra ocular lens) opacity, primary angle-closure glaucoma, and normal condition) and indicated by likelihood. (**A**) Slit-lamp microscope image analyzed as ‘normal condition’ by CorneAI. (**B**) Smartphone camera image of immunological keratitis misclassified as ‘cataract/IOL opacity’ by CorneAI. (**C**) Smartphone camera image of cataract/IOL opacity misclassified as ’normal condition’ by CorneAI.

Forty ophthalmologists (20 board-certified specialists and 20 residents) were first asked to classify the images without CorneAI (First test). After 2–4 weeks, they were asked to classify the same images again using CorneAI (Second test) (Fig. 3). In the classification with CorneAI support, CorneAI can indicated 9 classifications with likelihood values (Fig. 4). For each image, the sum of the likelihoods for the nine classifications displayed by CorneAI does not equal 100; each of the nine classifications is assigned a likelihood ranging from 0 to 100. We compared the performance of classification between with and without CorneAI support, and between board-certified specialists and residents. Three corneal specialists (H.M, Y.U, and T.Y) who were aware of the classification results were excluded from the survey. Additionally, the time required to complete the classification of 100 images were also assessed.

### Statistics

Statistical analyses were performed using JMP16 software (SAS Institute, Cary, NC, USA). The Wilcoxon rank sum test was used to compare the accuracy of diagnosis and the time for CorneAI and 40 ophthalmologists to complete classification of 100 images with and without CorneAI support. We also compared the results between board-certified specialists and residents, between smartphone and slit-lamp images, and among classifications. The sample size was determined using a Sample Size Calculator with an alpha of 5% and a power (1-β) of 0.80. Cases with any missing clinical data were excluded from the analysis in this study. Likewise, a student’s t-test was performed for sensitivity analysis, and similar results were obtained. *P* < 0.05 was considered statistically significant.

## Results

**Fig. 5:**
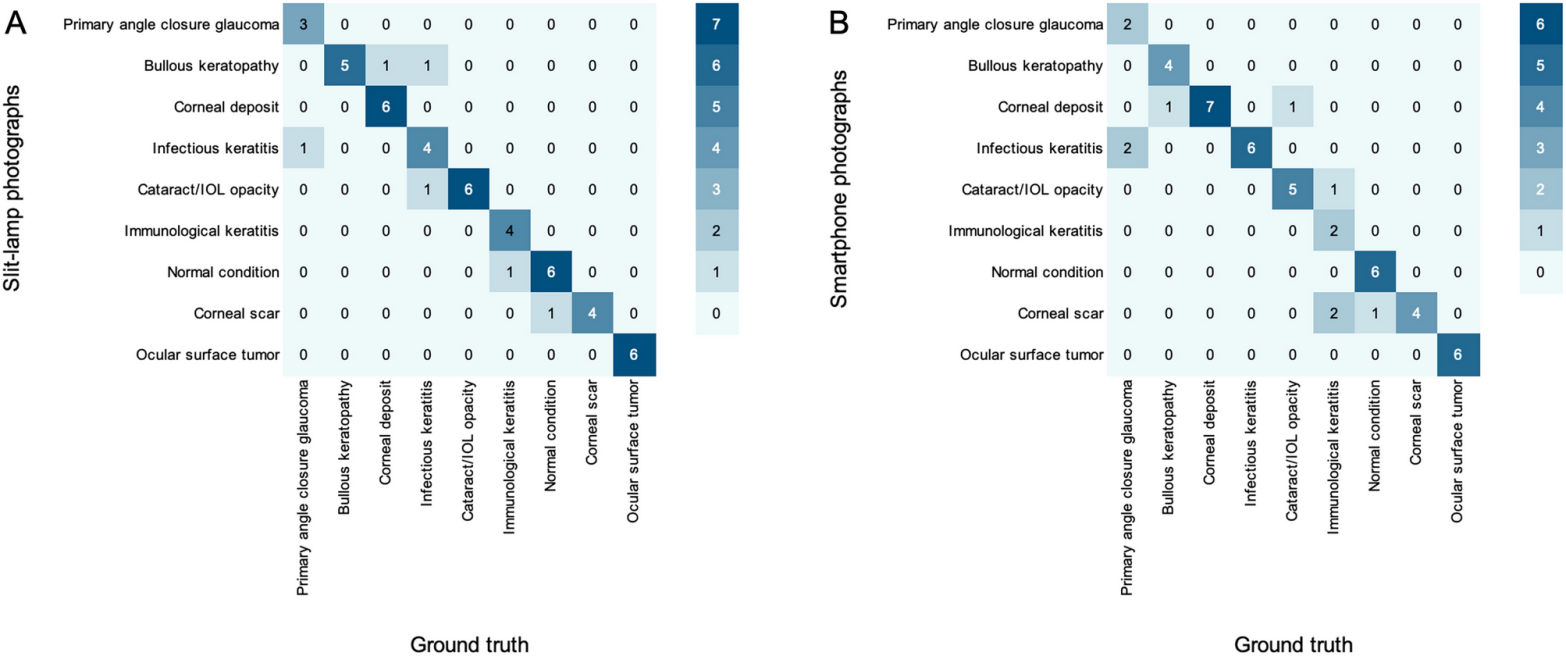
CorneAI classified 9 types of anterior segment diseases and the corresponding accuracy rates are represented using a color map. (**A**) The accuracy table for smartphone images is evaluated using CorneAI. (**B**) The accuracy of slit-lamp images is assessed using CorneAI. (IOL: intra ocular lens).

Of the100 images (i.e., a smartphone and slit-lamp images for each of 50 eyes) included in this study, CorneAI achieved a classification accuracy of 86.0%. There was no significant difference in the percentage of correct responses by CorneAI between smartphone (84.0%) and slit-lamp images (88.0%) (*P* = 0.95). CorneAI demonstrated 85.7% accuracy across all image types for normal conditions, 83.3% for smartphone images and 66.7% for slit-lamp images in infectious keratitis, and 60.0% for total images, 40.0% for smartphone images, and 80.0% for slit-lamp images in immunological keratitis. It achieved 100% accuracy across all image types for corneal scar and ocular surface tumors. For corneal deposits, CorneAI achieved 92.9% for total images, 100% for smartphone images, and 85.7% for slit-lamp images. For bullous keratopathy, it demonstrated 90.0% for total images, 80.0% for smartphone images, and 100% for slit-lamp images. For cataract and IOL opacity, slit-lamp images achieved 100% accuracy, while smartphone images reached 83.3%. In primary angle-closure glaucoma, it achieved 62.5% for total images, 50.0% for smartphone images, and 75.0% for slit-lamp images (Table 1, Fig. 5).

**Table 1:** Number of correct answers and percentage of correct answers with 9 classifications by CorneAI.

	Smartphone + Slit-lamp images (N = 100)		Smartphone images (N = 50)		Slit-lamp images (N = 50)	
	Correct answer/total	%	Correct answer/total	%	Correct answer/total	%
Normal condition	12/14	85.7	6/7	85.7	6/7	85.7
Infectious keratitis	10/12	83.3	6/6	100	4/6	66.7
Immunological keratitis	6/10	60.0	2/5	40.0	4/5	80.0
Cornear scaring	8/8	100	4/4	100	4/4	100
Corneal deposition	13/14	92.9	7/7	100	6/7	85.7
Bullous keratopathy	9/10	90.0	4/5	80.0	5/5	100
Ocular surface tumor	12/12	100	6/6	100	6/6	100
Cataract/IOL opacity	11/12	91.7	5/6	83.3	6/6	100
Primary angle-closure glaucoma	5/8	62.5	2/4	50.0	3/4	75.0

*IOL* intraocular lens.

**Fig. 6:**
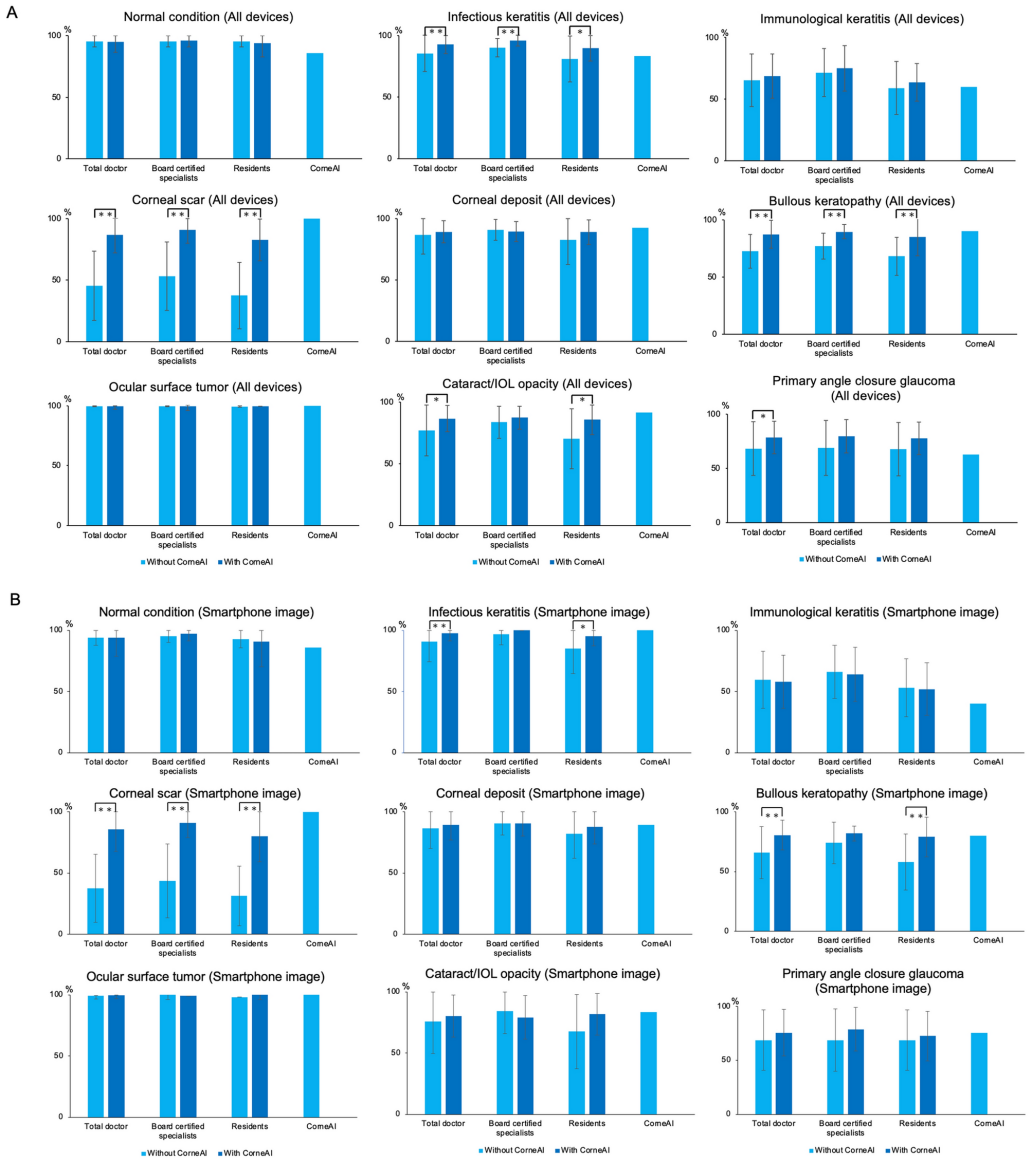

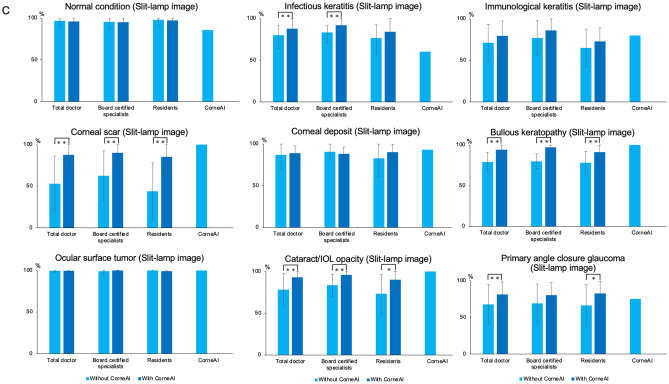
The change in the accuracy of ophthalmologists before and after with CorneAI support in the classification of 9 categories. (**A**) is the change in accuracy of 9 classifications combining smartphone and slit-lamp image with CorneAI support. In each classification, the accuracy of ophthalmologists significantly increased with CorneAI support for infectious keratitis, corneal scar, bullous keratopathy, cataract/IOL (intra ocular lens) opacity, and primary angle closure glaucoma (*: *P* < 0.05, **: *P* < 0.01). (**B**) is the change in accuracy of 9 classifications only smartphone image with CorneAI support. In each classification, the accuracy of ophthalmologists significantly increased with CorneAI support for infectious keratitis, corneal scar, bullous keratopathy (*: *P* < 0.05, **: *P* < 0.01). (**C**) is the change in accuracy of 9 classifications only slit-lamp image with CorneAI support. In each classification, the accuracy of ophthalmologists significantly increased with CorneAI support for infectious keratitis, corneal scar, bullous keratopathy, cataract/IOL opacity, and primary angle closure glaucoma (*: *P* < 0.05, **: *P* < 0.01).

The overall classification accuracy of ophthalmologists was 79.2 ± 7.9% (average ± SD) without CorneAI support. There was no significant difference in the accuracy between the images captured with smartphone cameras or slit-lamp microscope (78.8 ± 23.2% and 81.6 ± 21.8%, respectively; *P* = 0.54). The accuracy of board-certified specialists was significantly higher than that of residents (82.8 ± 5.4% vs. 75.6 ± 8.4%, *P* = 0.0034). Similarly, when compared stratifying the images based on the image capturing methods, there was no significant difference in the accuracy between board-certified specialists and residents in smartphone images (82.8 ± 22.8% and 75.1 ± 25.5%, respectively; *P* = 0.12) and in slit-lamp images (84.1 ± 20.9% and 78.3 ± 24.8%, respectively; *P* = 0.23). The accuracy for the nine classifications is shown in Fig. 6A–C.

With CorneAI support, the overall classification accuracy by ophthalmologists improved significantly from 79.2 ± 7.9% to 88.8 ± 5.3% (*P* < 0.001). The accuracy also significantly improved from 78.8 ± 23.2% to 85.8 ± 22.8% with CorneAI support in smartphone images, and from 81.6 ± 21.8% to 89.2 ± 14.8% in slit-lamp images (both *P* < 0.001).

The images with an accuracy of less than 70% without CorneAI support included 26 images out of 100, which included 2 images of infectious keratitis (1 smartphone and 1 slit-lamp images), 5 images of immunological keratitis (3 smartphone and 2 slit-lamp images), 8 images of scarring (4 smartphone and 4 slit-lamp images), 3 images of bullous keratopathy (1 smartphone and 2 slit-lamp images), and 5 images of primary angle-closure glaucoma (2 smartphone and 3 slit-lamp images). With CorneAI assistance, the accuracy by ophthalmologists was significantly improved in 19 out of these 26 images. The remaining 7 images with the accuracy lower than 70% included 1 image of infectious keratitis (a slit-lamp image), 2 images of immunological keratitis (a smartphone image), 1 image of bullous keratopathy (a smartphone image), and 3 images of primary angle-closure glaucoma (2 smartphone and 1 slit-lamp images). Interestingly, CorneAI provided correct suggestions in 19 images, leading to an improvement in accuracy, and incorrect suggestions in 7 images without the improvement. Additionally, CorneAI indicated likelihoods for more than two categories in 6 out of 7 images without an accuracy improvement. The multiple suggestions by CorneAI included 3 suggestions of lens opacities (1 smartphone and 2 slit-lamp images) and 3 images of immunological keratitis (2 smartphone images and 1 slit-lamp images) as shown in Table 2. Table 2 presents the accuracy of the corresponding images with and without CorneAI assistance.

**Table 2:** Questions for which CorneAI showed two or more likelihoods and the change in the percentage of correct answers by ophthalmologists who looked at the results.

No	Real ansewer	Device	CorneAI answer (in order of likehood)						Without CorneAI (%)	With CorneAI (%)
			1st		2nd		3rd			
1	Cataract/IOL opacity	Smartphone	Bullus keratopacty	(0.352)	Corneal scaring	(0.185)	Cataract/IOL opacity	(0.105)	14.3	29.3
2	Cataract/IOL opacity	Slit-lamp	Corneal deposition	(0.432)	Normal condition	(0.206)	Cataract/IOL opacity	(0.183)	83.3	90.2
3	Cataract/IOL opacity	Slit-lamp	Cataract/IOL opacity	(0.505)	Normal condition	(0.439)	–	–	47.6	78
4	Immunological keratitis	Smartphone	Corneal scaring	(0.658)	Immunological keratitis	(0.10)	–	–	88.1	82
5	Immunological keratitis	Slit-lamp	Immunological keratitis	(0.537)	Infectious keratitis	(0.408)	–	–	50	73.2
6	Immunological keratitis	Smartphone	Cataract/IOL opacity	(0.723)	Normal condition	(0.174)	–	–	21.4	9.8

*IOL* intraocular lens.

**Fig. 7:**
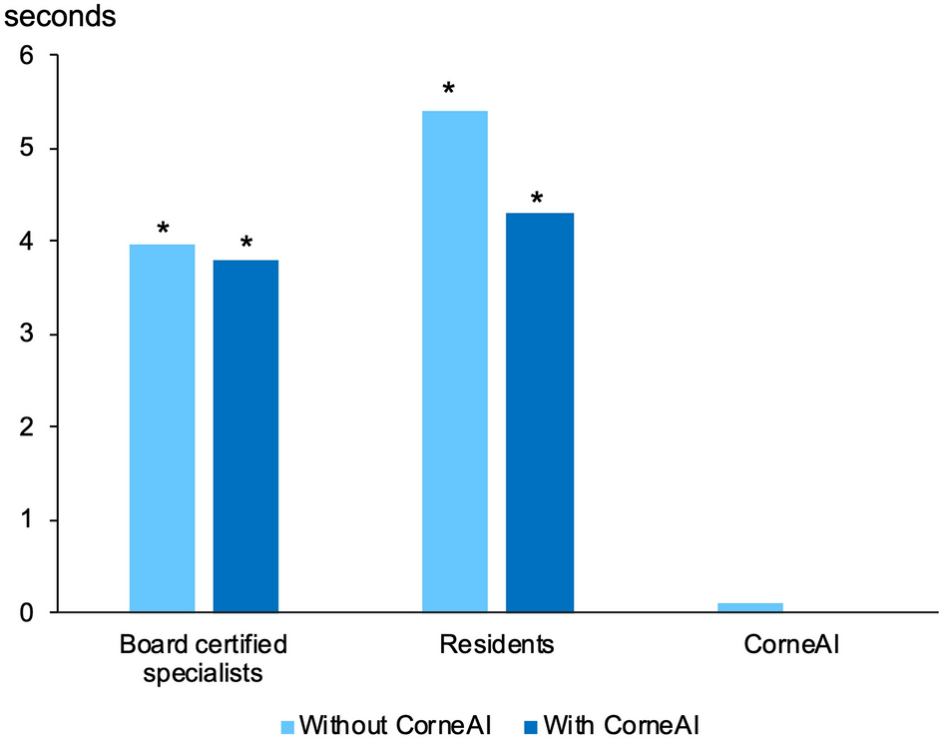
The time taken to classify 100 anterior segment eye diseases. Both board-certified specialists and residents completed the second survey with the use of CorneAI, in less time compared to the without CorneAI. However, there was no significant difference. In either case, the time taken for CorneAI to classify the 100 images were significantly shorter than that of ophthalmologists. *: *P* < 0.001 vs CorneAI.

The average time required to complete 100 classifications was 4.0 ± 1.4 s/image for board-certified specialists, and 5.4 ± 4.3 s/image for residents without CorneAI support, which was reduced to 3.8 ± 1.4 s/image in board-certified specialists, and 4.3 ± 3.2 s/image in resident (P = 0.74, P = 0.17, respectively) with CorneAI. The responses from CorneAI took approximately 0.1 s (Fig. 7). In all cases, the response time of CorneAI was significantly shorter (*P* < 0.001, respectively).

## Discussion

The results of this study demonstrate the potential of AI-assisted classification using CorneAI to improve the accuracy of ophthalmologist’s interpretations of anterior segment color images. The improved classification accuracy with AI assistance indicates the valuable role AI can play in clinical practice, particularly in managing various anterior segment diseases in hospitals or clinics. In the field of medical imaging, AI has demonstrated its capabilities in detection lung cancer in conventional X-rays and CT scans^11^, as well as identifying breast cancer through mammography^12^. Additionally, AI has shown promising results in recognition cartilage and ligament injuries in orthopedics^13^. In ophthalmology, AI diagnosis of diabetic retinopathy is feasible^14^, and an AI-equipped fundus camera has received approval from the Food and Drug Administration^15^. While some papers have compared AI performance with healthcare professionals using the same sample model, these reports often focus solely on AI performance without discussing its impact on physician diagnosis^16^. Our study is significant as the first paper to discuss the influence on ophthalmologists when presented with answers generated by AI. The research suggests an improvement in the diagnostic accuracy of ophthalmologists with AI assistance, indicating the effectiveness of AI support. Moreover, considering the potential application of smartphone-based approaches, there is a possibility of extending these benefits to remote healthcare settings.

Importantly, despite being primarily trained on slit-lamp images, CorneAI showed robust performance when applied to smartphone images, indicating its versatility and generalizability^4^. Corneal diseases are rare and are characterized by their diverse nature^17^. Among corneal diseases, infection keratitis ranks as the fifth leading cause of blindness worldwide, posing the risk of blindness even in younger individuals^18–20^. The accuracy for infectious keratitis, corneal scar, bullous keratopathy, cataract/IOL opacity, and primary angle-closure glaucoma infection, significantly increased with AI support, whether using a smartphone or slit-lamp. Particularly noteworthy is the ability of AI to accurately diagnose infections, which is crucial for selecting appropriate eye drops. Early detection and timely medical intervention are vital in maintaining good vision and positively influencing disease progression^20,21^. While there are existing reports on AI diagnosis for infectious keratitis^17,22,23^, there is a lack of reports on AI diagnosis for other anterior segment diseases such as immunological keratitis, dystrophies, and primary angle-closure glaucoma. CorneAI has demonstrated the ability to diagnose conditions such as cataracts and primary angle-closure glaucoma, indicating its potential for various anterior segment diseases and showcasing versatility in its diagnostic capabilities^4^.

Moreover, previous studies have reported equivalent diagnostic capabilities between images taken with slit-lamp microscopes and smartphones with attachments^6–10,24,25^. In our study, although smartphone images were taken without attachments, their diagnostic accuracy was found to be comparable to that of slit images. This suggests that patients and non-ophthalmologists can use their smartphones to take high-quality images suitable for teleophthalmology. However, it might be essential to emphasize the importance of providing proper training and instructions to patients and non-ophthalmologists for capturing these images accurately. These findings suggest that AI holds significant potential to enhance the quality of ophthalmological diagnoses and reduce the risk of misdiagnosis. The misdiagnosed images by CorneAI were analyzed in Fig. 4B (smartphone) and 4C (slit-lamp). Figure 4B represents a case of immunological keratitis with a lower corneal ulcer. However, the reason for its misclassification in the smartphone image is likely due to the smartphone’s illumination resembling that of a cataract. Figure 4C represents a cataract, but the morphology of the lens opacity resembled the slit-lamp light pattern, leading to its misclassification as a normal condition. Since smartphone images have not yet been incorporated into CorneAI training, the accuracy for these images was lower. We are currently developing an AI trained with smartphone images and preparing a manuscript for publication. Figure 4C features an atypical lens opacity that resembled the slit-lamp illumination pattern. CorneAI has primarily been trained on typical disease images, which may account for the misclassification in atypical cases. Re-training the AI with atypical images is expected to improve its diagnostic accuracy. Additionally, while CorneAI was trained using images taken with a slit-lamp, we believe there is room for further improvement in accuracy by training it with images taken by smartphones.

We also investigated the time required to complete 100 questions. The reduced response time with CorneAI support is expected to expedite the process and alleviate the workload for ophthalmologists. In human tasks, there is a potential for decreased concentration and accuracy when performing repetitive tasks. In this context, employing AI can lead to time savings and help streamline work processes, thereby enhancing efficiency. Reports from fields such as orthopedics, radiology, and information technology have indicated that AI assistance reduces the time needed to interpret X-ray images, aligning with the findings of our study^26–28^. Ophthalmologists expressed concerns about the extended time required for diagnosis when using CorneAI, as they need to consider not only the patient’s images but also the information provided by the AI. However, the results indicated an increase in diagnostic accuracy and a trend toward shorter response times. This suggests that, in the context of image diagnosis, AI could effectively reduce misdiagnoses efficiently. Even in cases where CorneAI indicated two or more likelihoods, an improvement in accuracy was observed in 4 out of 6 cases. This implies that AI assistance may provide a valuable reference in narrowing down the potential diagnoses for anterior segment diseases. The potential for time reduction with AI assistance has been previously reported^29,30^, but our study represents the first report demonstrating a reduction in response time. While previous reports often focused on AI handling extensive data processing, which could be challenging for healthcare professionals^31^, our study suggests that CorneAI support can efficiently aid ophthalmologists in diagnosis, laying the groundwork for the future development of AI in ophthalmology.

Finally, our study has several limitations that should be considered when interpreting the results. Firstly, the ophthalmologists who participated in this study were affiliated with university hospitals, which may distinguish them from those working in general hospitals or clinics. Secondly, the time allowed for ophthalmologists to answer the same set of questions varied from 2 to 4 weeks. According to Ebbinghaus’ forgetting curve, humans tend to forget around 76% of what they’ve learned after one day and approximately 80% after one week^32^. In our study, after the first test, no answers were provided to the ophthalmologists, and there was a gap of over two weeks was introduced before the second test. Additionally, the order of questions was randomly shuffled for the second test. While, it is believed that the likelihood of the ophthalmologists remembering the answers from the first test is low, it cannot be entirely ruled out, presenting a limitation of this study. Ultimately, the diagnosis of diseases must be made by physicians, and AI serves only as a diagnostic support tool. Since there is a possibility that AI may make diagnostic errors, it is essential for physicians utilizing AI to possess accurate knowledge. Even in this era of medical innovation brought about by AI, clinical experience remains indispensable for unlocking its true potential. Physicians must continue their efforts to refine their expertise, as we have reported^33^. In this study, CorneAI achieved an accuracy rate of 86%, and even at this level of accuracy, ophthalmologists’ diagnostic accuracy improved with AI support. Enhancing the performance of AI has the potential to further increase the diagnostic accuracy of ophthalmologists. Therefore, improving the performance of AI remains one of our critical objectives.

## Conclusion

In conclusion, our study highlights the potential of AI to improve the accuracy of interpreting anterior segment color images by ophthalmologists. Our findings indicate that AI support can be advantageous for non-specialists and when utilizing smartphone imaging devices. The CorneAI support for anterior segment images not only improved the accuracy of ophthalmologists but also showcased the effectiveness of using images taken with smartphones without attachments. We believed that CorneAI, as a second-reader type program, is clinically feasible and could serve as a valuable tool in telemedicine applications.

## Supplementary Information

Supplementary Information.

## Data Availability

The datasets used and/or analysed during the current study available from the corresponding author on reasonable request.

## Abbreviations

AI: Artificial intelligence
IOL: Intra ocular lens
