# Supplementary figures and images for: Artificial intelligence support improves diagnosis accuracy in anterior segment eye diseases
Source: Sci Rep. 2025 Feb 11;15:5117. doi: 10.1038/s41598-025-89768-6 (PMC11814138; doi:10.1038/s41598-025-89768-6)

## Slide 1
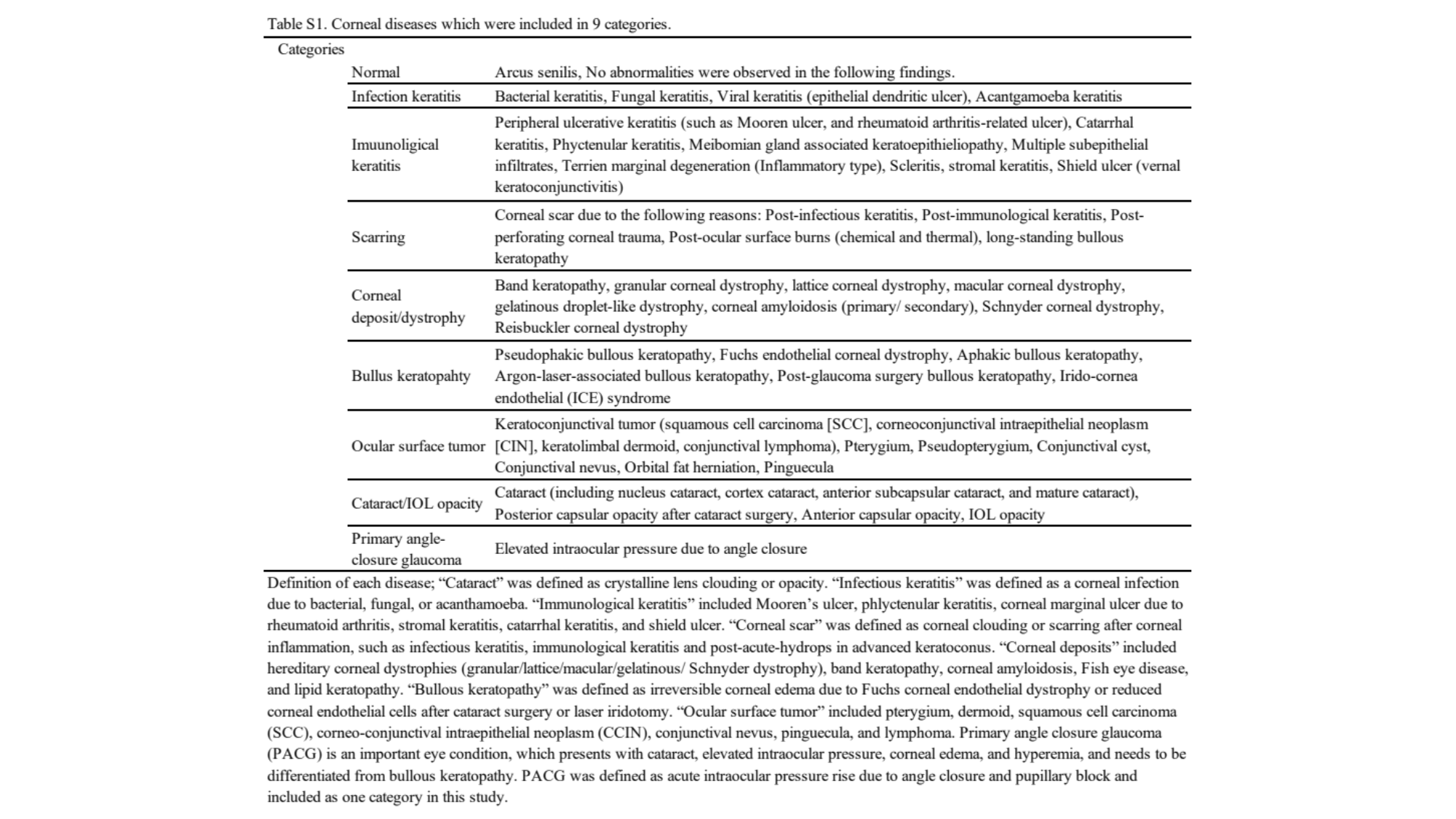

Supplement: Supplementary file 1 — Supplementary Information. [file 41598_2025_89768_MOESM1_ESM.pptx]
